# Supplementary material for: A comprehensive analysis of Atlantic salmon gonad and pituitary transcriptomes identifies novel players in sexual maturation
Source: BMC Genomics. 2025 Sep 26;26:818. doi: 10.1186/s12864-025-11954-7 (PMC12465306; doi:10.1186/s12864-025-11954-7)
Supplement: Supplementary file 3 — Additional file 3. Supplementary_file3_candidate_maturation_related_newly_characterized_gene.docx. Maturation related candidate newly characterized genes. 𝜏 value of each loci showing its tissue specificity * indicates loci upregulated in a single tissue, it: immature testis, mt: mature testis, ov: ovary, pt: pituitary. [file 12864_2025_11954_MOESM3_ESM.docx]

**Maturation related candidate newly characterized genes.**

𝜏 value of each loci showing its tissue specificity * indicates loci upregulated in a single tissue, it: immature testis, mt: mature testis, ov: ovary, pt: pituitary

| Module | Geneid | Location | 𝜏 | Tissue | Gene symbol | GO pathway | Reference |
| --- | --- | --- | --- | --- | --- | --- | --- |
| turquoise | MSTRG.19353 | 14:29452500-29454393 | 0.97 | it | *foxq1* | sequence-specific DNA binding | [1] |
| turquoise | MSTRG.36976 | 2:45587769-45591283 | 0.98 | it | *pou3f2* | sequence-specific DNA binding | [2] |
| turquoise | MSTRG.43887 | 22:26980917-26999946 | 0.78 | it | *map4k1* | intracellular signal transduction | [3, 4] |
| turquoise | MSTRG.47909 | 24:42890640-42899662 | 0.93 | it | *chrna2* | transmembrane signaling receptor activity;transmembrane transporter activity | [5] |
| turquoise | MSTRG.50203 | 26:22274896-22279093 | 0.94 | it | *mc1r* | intracellular signal transduction;transmembrane signaling receptor activity | [6, 7] |
| turquoise | MSTRG.51093 | 26:52745846-52752194 | 0.74 | it | *ttn* | intracellular signal transduction;protein tyrosine kinase activity;actin filament binding;myosin complex | [8] |
| turquoise | MSTRG.66318 | 6:43972990-43975586 | 0.75 | it | *hoxb5* | sequence-specific DNA binding | [9] |
| turquoise | MSTRG.71253 | 9:17006614-17024991 | 0.82 | it | *gjb3* | transmembrane transporter activity | [10] |
| turquoise | MSTRG.7678 | 10:101440111-101451026 | 0.89 | it | *nfic* | sequence-specific DNA binding | [11] |
| turquoise | MSTRG.77909 | CAJNNT020001543.1:98775-102564 | 0.76 | it | *map4k1* | intracellular signal transduction | [3, 4] |
| turquoise | MSTRG.9788 | 11:54024351-54027406 | 0.88 | it | *cebpa* | sequence-specific DNA binding | [12] |
| turquoise | MSTRG.77236 | CAJNNT020001308.1:163721-195053 | 0.39 | pt* | *wnk1* | intracellular signal transduction | [13] |
| turquoise | MSTRG.30515 | 17:84363103-84405843 | 0.40 | pt* | *wnk1* | intracellular signal transduction |  |
| blue | MSTRG.14389 | 12:78943353-78949043 | 0.94 | mt | *nfic* | sequence-specific DNA binding | [11] |
| blue | MSTRG.31161 | 18:17331978-17332733 | 0.89 | mt | *nfic* | sequence-specific DNA binding |  |
| blue | MSTRG.41134 | 20:73029699-73038125 | 1.00 | mt | *nfic* | sequence-specific DNA binding |  |
| blue | MSTRG.17899 | 13:96185476-96240120 | 0.48 | mt | *clip1* | cytoskeleton;cytoskeleton organization;actin binding;cell projection;plasma membrane bounded cell projection;neuron projection;microtubule binding;intracellular protein transport | [14] |
| blue | MSTRG.23444 | 15:53950937-53955657 | 1.00 | mt* | *exoc3* | cell projection;plasma membrane bounded cell projection | [15, 16] |
| blue | MSTRG.23516 | 15:58049402-58059974 | 1.00 | mt* | *phc1* | regulation of cell cycle process;cellular response to cytokine stimulus | [17] |
| blue | MSTRG.30638 | 17:86893690-86896024 | 1.00 | mt | *phc1* | regulation of cell cycle process;cellular response to cytokine stimulus |  |
| blue | MSTRG.25955 | 16:28008461-28014267 | 1.00 | mt* | *pop-1* | kinase binding;sequence-specific DNA binding | [18] |
| blue | MSTRG.33633 | 19:29102282-29104208 | 0.55 | pt | *c12orf55 (CFAP54)* | cilium movement involved in cell motility;cilium assembly;cytoskeleton;axoneme;cell projection assembly;cilium;cell projection;plasma membrane bounded cell projection;plasma membrane bounded cell projection assembly;cilium organization;microtubule-based movement | [19] |
| blue | MSTRG.38636 | 2:86521879-86534355 | 1.00 | mt* | *haus7* | cilium assembly;cytoskeleton;cytoskeleton organization;cell projection assembly;plasma membrane bounded cell projection assembly;regulation of cell cycle process;cilium organization | [20] |
| blue | MSTRG.38830 | 2:88262406-88263034 | 1.00 | mt* | *hmgb1* | lipid binding;cytoskeleton organization;cell projection;plasma membrane bounded cell projection;positive regulation of chemotaxis;apoptotic process;membrane raft;neuron projection;GO:0051272;sequence-specific DNA binding;cellular response to cytokine stimulus | [21, 22] |
| blue | MSTRG.43694 | 22:19570003-19570939 | 1.00 | mt | *odc1* | carboxy-lyase activity;carbon-carbon lyase activity | [23, 24] |
| blue | MSTRG.48559 | 25:17958106-17963460 | 0.95 | mt | *arl4c* | cell projection;plasma membrane bounded cell projection | [25] |
| blue | MSTRG.50594 | 26:35236466-35240379 | 1.00 | mt | *dvl2* | transmembrane receptor protein tyrosine kinase signaling pathway;lipid binding;cilium assembly;cytoskeleton;cytoskeleton organization;regulation of actin cytoskeleton organization;axon guidance;cell projection assembly;cilium;kinase binding;cell projection;plasma membrane bounded cell projection;positive regulation of chemotaxis;plasma membrane bounded cell projection assembly;regulation of cell cycle process;cilium organization;GO:0051272 | [26, 27] |
| blue | MSTRG.73428 | 9:92393500-92396027 | 1.00 | mt | *rab11fip5* | cytoskeleton;kinase binding;cellular response to cytokine stimulus | [28] |
| blue | MSTRG.81613 | CAJNNT020003506.1:50387-57638 | 0.52 | mt* | *dnah7* | cilium assembly;cytoskeleton;cytoskeleton organization;axoneme;cell projection assembly;cilium;minus-end-directed microtubule motor activity;dynein intermediate chain binding;dynein light intermediate chain binding;cell projection;plasma membrane bounded cell projection;plasma membrane bounded cell projection assembly;dynein complex;cilium organization;microtubule-based movement | [29] |
| blue | MSTRG.82129 | CAJNNT020004027.1:40133-46756 | 0.51 | mt | *dnah6* | cilium assembly;cytoskeleton;axoneme;cell projection assembly;cilium;minus-end-directed microtubule motor activity;dynein intermediate chain binding;dynein light intermediate chain binding;cell projection;plasma membrane bounded cell projection;plasma membrane bounded cell projection assembly;dynein complex;cilium organization;microtubule-based movement | [30] |
| blue | MSTRG.9377 | 11:35108607-35112465 | 1.00 | mt | *dvl2* | transmembrane receptor protein tyrosine kinase signaling pathway;lipid binding;cilium assembly;cytoskeleton;cytoskeleton organization;regulation of actin cytoskeleton organization;axon guidance;cell projection assembly;cilium;kinase binding;cell projection;plasma membrane bounded cell projection;positive regulation of chemotaxis;plasma membrane bounded cell projection assembly;regulation of cell cycle process;cilium organization;GO:0051272 | [27] |
| brown | MSTRG.26507 | 16:45185343-45187305 | 0.52 | ov* | *ier5* | regulation of transcription by RNA polymerase II | [31] |
| brown | MSTRG.39497 | 20:20812801-20814199 | 0.52 | ov* | *anapc5* | ubiquitin-protein transferase activity | [32] |
| green | MSTRG.33275 | 19:9749110-9754623 | 1.00 | pt | *amer2* | phospholipid binding | [33] |
| green | MSTRG.64355 | 5:85319395-85322944 | 1.00 | pt* | *nbeal2* | secretory granule | [34] |
| green | MSTRG.66383 | 6:45618184-45621858 | 1.00 | pt* | *gh1* | hormone activity;secretory granule;sodium ion transport | [35] |
| green | MSTRG.81880 | CAJNNT020003707.1:118842-162475 | 0.99 | pt | *ugt8* | UDP-glycosyltransferase activity | [36] |
| yellow | MSTRG.49132 | 25:39104782-39107074 | 0.82 | pt | *slc3a2* | calcium ion transmembrane transport;sodium ion transport;transmembrane transporter activity | [37] |
| yellow | MSTRG.51044 | 26:51076808-51080606 | 1.00 | pt | *pten* | protein tyrosine phosphatase activity;protein dephosphorylation;dephosphorylation;chemical synaptic transmission;intracellular signal transduction;phosphatase activity;actin binding;transmembrane receptor protein tyrosine kinase signaling pathway;postsynaptic membrane | [38] |
| yellow | MSTRG.57095 | 3:58358084-58361260 | 0.87 | pt | *krt12* | signaling receptor activity;transmembrane signaling receptor activity;protein dimerization activity | [39] |
| yellow | MSTRG.67178 | 6:72434470-72437104 | 0.91 | pt | *gpr6* | signaling receptor activity;transmembrane signaling receptor activity;intracellular signal transduction | [40] |
| yellow | MSTRG.79860 | CAJNNT020002330.1:2300-8334 | 0.88 | pt | *cpe* | neurexin family protein binding;chemical synaptic transmission | [41] |

**Reference**

1. Liu Z, Yuan M, Meng X, Bie H, Yao S. Identification of testicular Foxq1 as a critical modulator of lactate metabolism in mouse Sertoli cells. Histochem Cell Biol. 2021;156:227–37.

2. Zini A, Mielnik A, Schlegel PN. POU-domain gene expression during spermatogenesis. World J Urol. 1996;14:274–7.

3. Chuang H-C, Wang X, Tan T-H. Chapter Seven - MAP4K Family Kinases in Immunity and Inflammation. In: Alt FW, editor. Advances in Immunology. Academic Press; 2016. p. 277–314.

4. Li MWM, Mruk DD, Cheng CY. Mitogen-activated protein kinases in male reproductive function. Trends Mol Med. 2009;15:159–68.

5. Pei S, Wang Z, Liu Y, Xu Y, Bai J, Li W, et al. Transcriptomic analysis of the HPG axis-related tissues reveals potential candidate genes and regulatory pathways associated with testicular size in Hu sheep. Theriogenology. 2024;216:168–76.

6. Mogil JS, Wilson SG, Chesler EJ, Rankin AL, Nemmani KVS, Lariviere WR, et al. The melanocortin-1 receptor gene mediates female-specific mechanisms of analgesia in mice and humans. Proc Natl Acad Sci. 2003;100:4867–72.

7. Navarro S, Crespo D, Schulz RW, Ge W, Rotllant J, Cerdá-Reverter JM, et al. Role of the Melanocortin System in Gonadal Steroidogenesis of Zebrafish. Anim Open Access J MDPI. 2022;12:2737.

8. Bai D-P, Chen Y, Hu Y-Q, He W-F, Shi Y-Z, Fan Q-M, et al. Transcriptome analysis of genes related to gonad differentiation and development in Muscovy ducks. BMC Genomics. 2020;21:438.

9. MacLean JA, Hu Z, Welborn JP, Song H-W, Rao MK, Wayne CM, et al. The RHOX Homeodomain Proteins Regulate the Expression of Insulin and Other Metabolic Regulators in the Testis*. J Biol Chem. 2013;288:34809–25.

10. Plum A, Hallas G, Willecke K. Expression of the mouse gap junction gene Gjb3 is regulated by distinct mechanisms in embryonic stem cells and keratinocytes. Genomics. 2002;79:24–30.

11. Davila RA, Spiller C, Harkins D, Harvey T, Jordan PW, Gronostajski RM, et al. Deletion of NFIX results in defective progression through meiosis within the mouse testis. Biol Reprod. 2022;106:1191.

12. Yao P-L, Lin Y-C, Richburg JH. Transcriptional suppression of Sertoli cell Timp2 in rodents following mono-(2-ethylhexyl) phthalate exposure is regulated by CEBPA and MYC. Biol Reprod. 2011;85:1203–15.

13. Chi RA, Xu X, Li J-L, Xu X, Hu G, Brown P, et al. WNK1 is required during male pachynema to sustain fertility. iScience. 2023;26:107616.

14. Larti F, Kahrizi K, Musante L, Hu H, Papari E, Fattahi Z, et al. A defect in the CLIP1 gene (CLIP-170) can cause autosomal recessive intellectual disability. Eur J Hum Genet. 2015;23:331–6.

15. Mikami N, Nguyen CLK, Osawa Y, Kato K, Ishida M, Tanimoto Y, et al. Deletion of Exoc7, but not Exoc3, in male germ cells causes severe spermatogenesis failure with spermatocyte aggregation in mice. Exp Anim. 2024;73:286–92.

16. Pisarek A, Pośpiech E, Heidegger A, Xavier C, Papież A, Piniewska-Róg D, et al. Epigenetic age prediction in semen – marker selection and model development. Aging. 2021;13:19145–64.

17. Steilmann C, Cavalcanti MCO, Bergmann M, Kliesch S, Weidner W, Steger K. Aberrant mRNA expression of chromatin remodelling factors in round spermatid maturation arrest compared with normal human spermatogenesis. Mol Hum Reprod. 2010;16:726–33.

18. Siegfried KR, Kimble J. POP-1 controls axis formation during early gonadogenesis in C. elegans. Development. 2002;129:443–53.

19. McKenzie CW, Craige B, Kroeger TV, Finn R, Wyatt TA, Sisson JH, et al. CFAP54 is required for proper ciliary motility and assembly of the central pair apparatus in mice. Mol Biol Cell. 2015;26:3140–9.

20. Li L, Sha Y-W, Su Z-Y, Mei L-B, Ji Z-Y, Zhang Q, et al. A novel mutation in HAUS7 results in severe oligozoospermia in two brothers. Gene. 2018;639:106–10.

21. Aslani F, Schuppe H-C, Guazzone VA, Bhushan S, Wahle E, Lochnit G, et al. Targeting high mobility group box protein 1 ameliorates testicular inflammation in experimental autoimmune orchitis. Hum Reprod Oxf Engl. 2015;30:417–31.

22. Zetterström CK, Strand M-L, Söder O. The high mobility group box chromosomal protein 1 is expressed in the human and rat testis where it may function as an antibacterial factor. Hum Reprod. 2006;21:2801–9.

23. Lee NKL, Skinner JPJ, Zajac JD, MacLean HE. Ornithine decarboxylase is upregulated by the androgen receptor in skeletal muscle and regulates myoblast proliferation. Am J Physiol Endocrinol Metab. 2011;301:E172-179.

24. Lefèvre PLC, Palin M-F, Murphy BD. Polyamines on the Reproductive Landscape. Endocr Rev. 2011;32:694–712.

25. Schürmann A, Koling S, Jacobs S, Saftig P, Krauβ S, Wennemuth G, et al. Reduced Sperm Count and Normal Fertility in Male Mice with Targeted Disruption of the ADP-Ribosylation Factor-Like 4 (Arl4) Gene. Mol Cell Biol. 2002;22:2761–8.

26. Fabijanovic D, Zunic I, Martic TN, Skenderi F, Serman L, Vranic S. The expression of SFRP1, SFRP3, DVL1, and DVL2 proteins in testicular germ cell tumors. APMIS Acta Pathol Microbiol Immunol Scand. 2016;124:942–9.

27. Sharma M, Castro-Piedras I, Simmons GE, Pruitt K. Dishevelled: a masterful conductor of complex Wnt signals. Cell Signal. 2018;47:52–64.

28. Cullis DN, Philip B, Baleja JD, Feig LA. Rab11-FIP2, an Adaptor Protein Connecting Cellular Components Involved in Internalization and Recycling of Epidermal Growth Factor Receptors*. J Biol Chem. 2002;277:49158–66.

29. Gao Y, Liu L, Shen Q, Fu F, Xu C, Geng H, et al. Loss of function mutation in DNAH7 induces male infertility associated with abnormalities of the sperm flagella and mitochondria in human. Clin Genet. 2022;102:130–5.

30. Shao Z-M, Zhu Y-T, Gu M, Guo S-C, Yu H, Li K-K, et al. Novel variants in DNAH6 cause male infertility associated with multiple morphological abnormalities of the sperm flagella (MMAF) and ICSI outcomes. Asian J Androl. 2023;26:91–8.

31. Yu X-P, Wu Y-M, Liu Y, Tian M, Wang J-D, Ding K-K, et al. IER5 is involved in DNA Double-Strand Breaks Repair in Association with PAPR1 in Hela Cells. Int J Med Sci. 2017;14:1292–300.

32. Han L, Chen Y, Li L, Ren C, Wang H, Wu X, et al. Increased mtDNA mutation frequency in oocytes causes epigenetic alterations and embryonic defects. Natl Sci Rev. 2022;9:nwac136.

33. Pfister AS, Tanneberger K, Schambony A, Behrens J. Amer2 Protein Is a Novel Negative Regulator of Wnt/β-Catenin Signaling Involved in Neuroectodermal Patterning*. J Biol Chem. 2012;287:1734–41.

34. Albers CA, Cvejic A, Favier R, Bouwmans EE, Alessi M-C, Bertone P, et al. Exome sequencing identifies NBEAL2 as the causative gene for gray platelet syndrome. Nat Genet. 2011;43:735–7.

35. Hull KL, Harvey S. Growth hormone. Endocrine. 2000;13:243–50.

36. Sikoki FD, Tubb RA, Curtis LR. Elevation of sex steroids and inhibition of PDP-glucuronyltransferase are out of phase during gonadal maturation in the common carp. Comp Biochem Physiol Part C Comp Pharmacol. 1989;92:267–72.

37. Kahlhofer J, Teis D. The human LAT1–4F2hc (SLC7A5–SLC3A2) transporter complex: Physiological and pathophysiological implications. Basic Clin Pharmacol Toxicol. 2023;133:459–72.

38. Neirijnck Y, Kühne F, Mayère C, Pavlova E, Sararols P, Foti M, et al. Tumor Suppressor PTEN Regulates Negatively Sertoli Cell Proliferation, Testis Size, and Sperm Production In Vivo. Endocrinology. 2019;160:387–98.

39. Hayashi Y, Call MK, Liu C-Y, Hayashi M, Babcock G, Ohashi Y, et al. Monoallelic Expression of Krt12 Gene during Corneal-type Epithelium Differentiation of Limbal Stem Cells. Invest Ophthalmol Vis Sci. 2010;51:4562–8.

40. Li Z, Jiang B, Cao B, Zhang Z, Zhang J, Li J, et al. Characterization of Four Orphan Receptors (GPR3, GPR6, GPR12 and GPR12L) in Chickens and Ducks and Regulation of GPR12 Expression in Ovarian Granulosa Cells by Progesterone. Genes. 2021;12:489.

41. Lynch DR, Braas KM, Hutton JC, Snyder SH. Carboxypeptidase E (CPE): immunocytochemical localization in the rat central nervous system and pituitary gland. J Neurosci. 1990;10:1592–9.
