# Supplementary material for: A comprehensive analysis of Atlantic salmon gonad and pituitary transcriptomes identifies novel players in sexual maturation
Source: BMC Genomics. 2025 Sep 26;26:818. doi: 10.1186/s12864-025-11954-7 (PMC12465306; doi:10.1186/s12864-025-11954-7)
Supplement: Supplementary file 4 — Additional file 4. ms_salmon_gnpt_rnaseq_supplementary_figures.docx. Figure S1. Two ROC curves for automatic detection of optimized CPS threshold and user specificity threshold. Red line indicates mRNA specificity. Blue line indicates lncRNA specificity. Figure S2. Soft threshold filtering. Scale-independence and mean connectivity of the network in different soft-threshold powers. The left panel displays the correlation of soft threshold with scale-free fit index. The right panel displays the influence of soft-threshold power on mean connectivity. Figure S3. WGCNA network construction (A), Sample dendrogram and tissue annotation (B), Cluster dendrogram. Figure S4. Chromosomal distribution of intergenic transcripts. Figure S5. Histogram of number of samples supporting intergenic transcripts. Figure S6. Boxplot of the number of samples supporting intergenic transcript per tissue type. Figure S7. Histogram of number of samples supporting intergenic transcripts in PRJNA380580. Figure S8. Histogram of number of support sample for the intergenic transcripts in PRJNA550414. Figure S9. A violin plot of RNA expression levels in transcripts per million (TPM) in four Atlantic salmon tissues. Figure S10. Violin plots of known/newly characterized protein-coding gene/lncRNA/lincRNA expression levels in transcripts per million (TPM) across four Atlantic salmon tissues. Figure S11. Tree plots of the top 30 significantly enriched GO terms in the GO enrichment analysis of yellow module. [file 12864_2025_11954_MOESM4_ESM.docx]

**Supplementary figures**


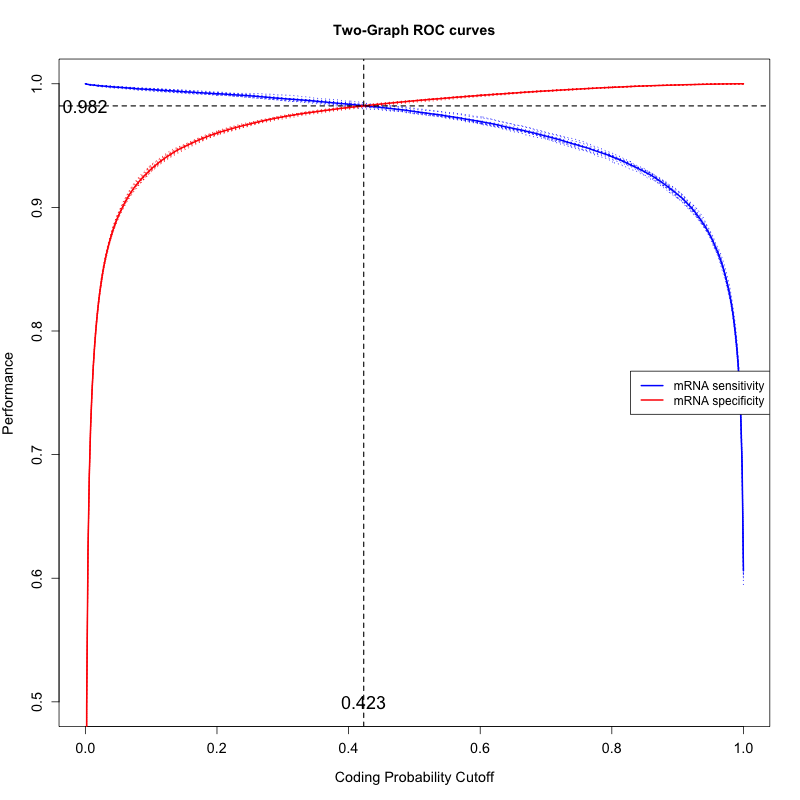


**Figure S1.** Two ROC curves for automatic detection of optimized CPS threshold and user specificity threshold. Red line indicates mRNA specificity. Blue line indicates lncRNA specificity.


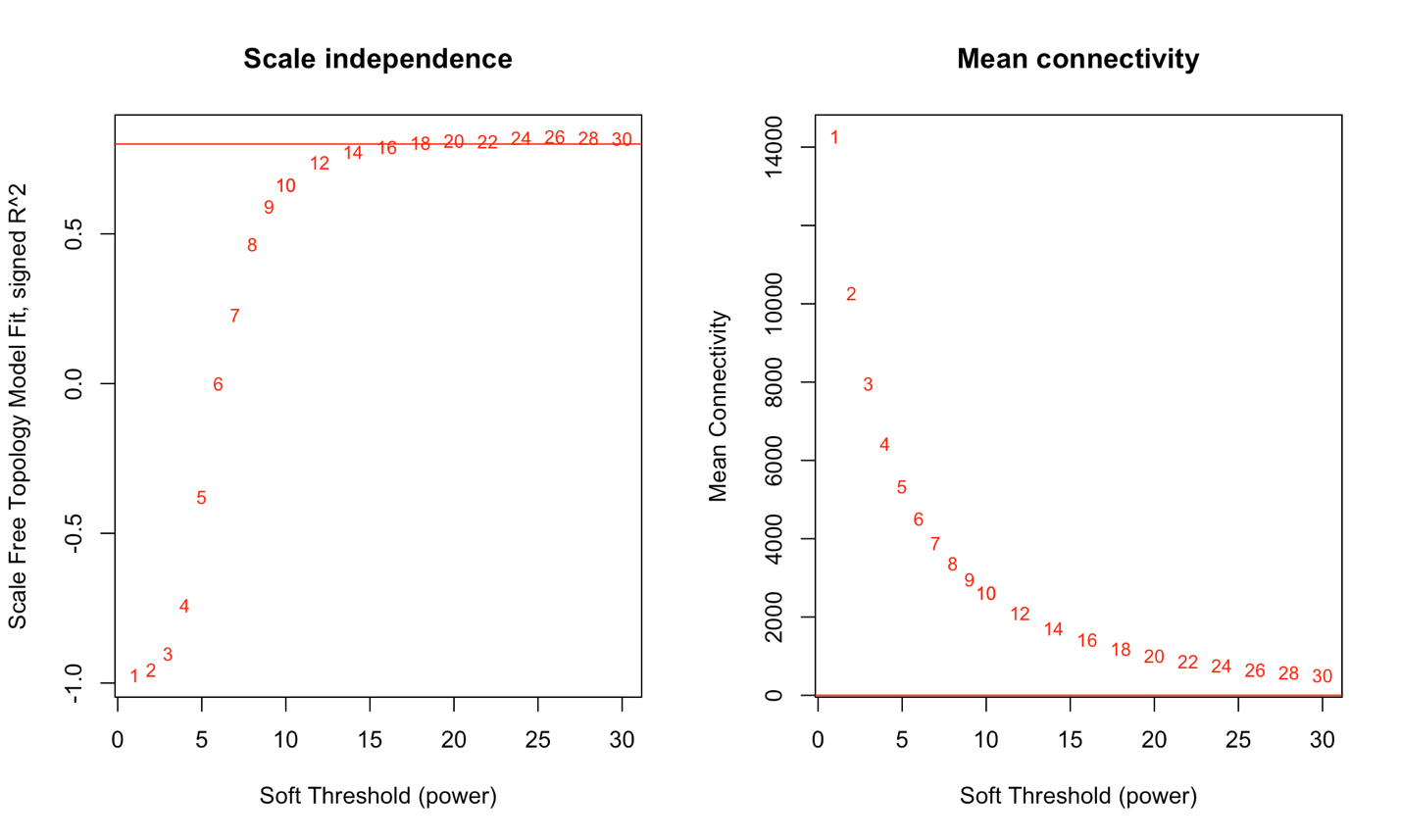


**Figure S2.** Soft threshold filtering. Scale-independence and mean connectivity of the network in different soft-threshold powers. The left panel displays the correlation of soft threshold with scale-free fit index. The right panel displays the influence of soft-threshold power on mean connectivity.

**Figure S3.** WGCNA network construction (A), Sample dendrogram and tissue annotation (B), Cluster dendrogram.

**Figure S4.** Chromosomal distribution of intergenic transcripts.


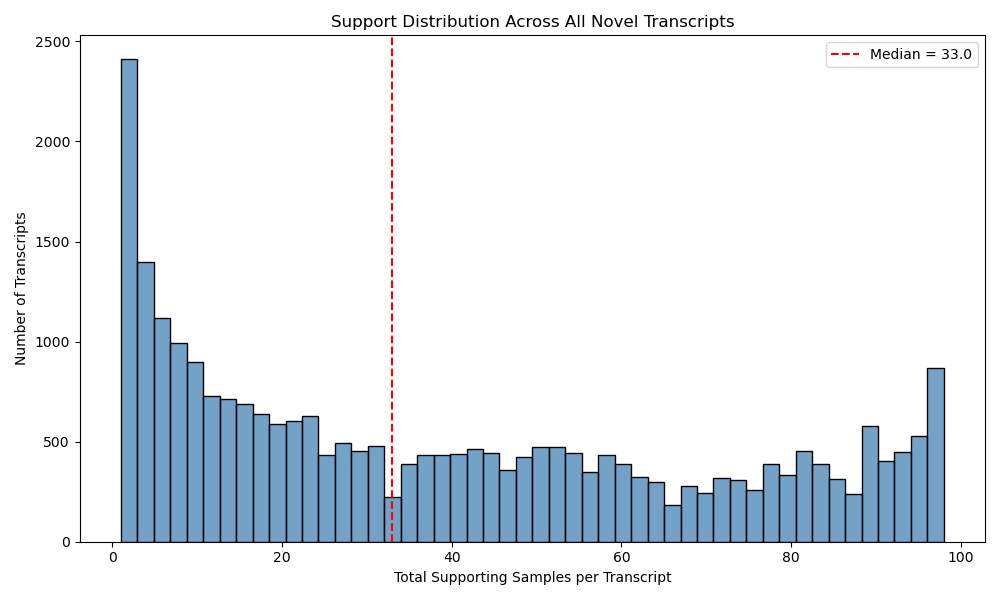


**Figure S5**. Histogram of number of samples supporting intergenic transcripts.


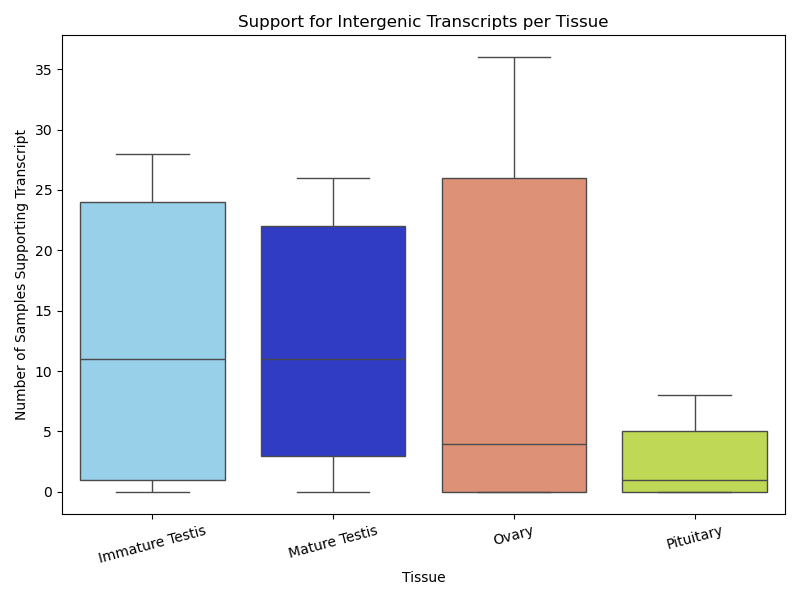


**Figure S6.** Boxplot of the number of samples supporting intergenic transcript per tissue type.


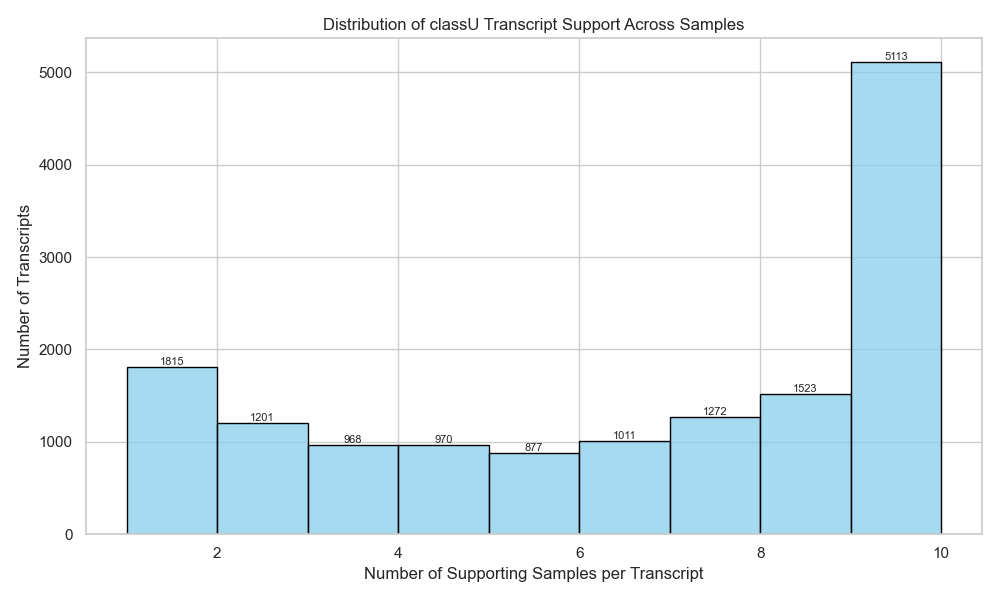


**Figure S7.** Histogram of number of samples supporting intergenic transcripts in PRJNA380580.


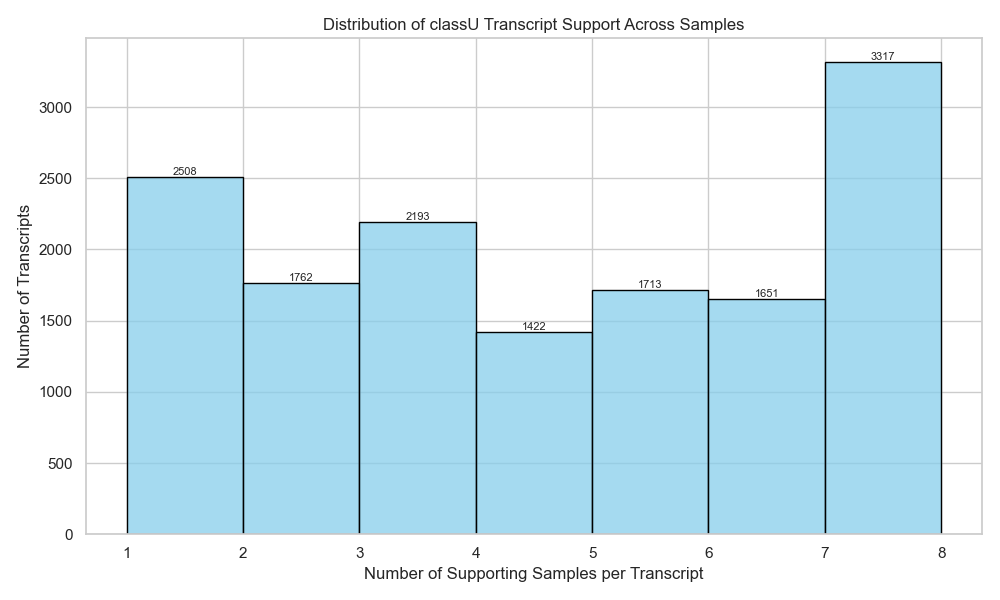


**Figure S8.** Histogram of number of support sample for the intergenic transcripts in PRJNA550414.

**Figure S9.** A violin plot of RNA expression levels in transcripts per million (TPM) in four Atlantic salmon tissues.

**Figure S10.** Violin plots of known/newly characterized protein-coding gene/lncRNA/lincRNA expression levels in transcripts per million (TPM) across four Atlantic salmon tissues.

**Figure S11.** Tree plots of the top 30 significantly enriched GO terms in the GO enrichment analysis of yellow module.
